# Germline Genetic Testing in Patients With Solid Malignant Tumors

**DOI:** 10.1001/jamanetworkopen.2025.22754

**Published:** 2025-07-23

**Authors:** Matthew E. Fine, Adina Estrin, Alissa Michel, Vicky Ro, Tarsha Jones, Meghna S. Trivedi, Katherine D. Crew

**Affiliations:** 1 Department of Medicine, Vagelos College of Physicians and Surgeons, Columbia University Irving Medical Center, New York, New York; 2 Mailman School of Public Health, Columbia University, New York, New York; 3 Now with GlaxoSmithKline, City, State; 4 Herbert Irving Comprehensive Cancer Center, Columbia University Irving Medical Center, New York, New York; 5 Christine E. Lynn College of Nursing, Florida Atlantic University, Boca Raton

## Abstract

This cohort study analyzes the clinical and demographic characteristics of patients with cancer who undergo germline genetic testing.

## Introduction

Current guidelines recommend germline genetic testing (GT) in patients with metastatic ERBB2-negative breast cancer, metastatic prostate cancer, all stages of epithelial ovarian cancer, and exocrine pancreatic cancer.^1,2^ With the arrival of targeted therapies such as poly(adenosine diphosphate–ribose) polymerase (PARP) inhibitors, results of GT now directly influence treatment decisions.^1,2^ The increasing demand for germline GT has led to the transition from genetic counselor–led GT to oncologist-led GT.^3,4^ However, access to GT services has lagged behind for individuals from racial and ethnic minoritized and socioeconomically disadvantaged groups.^5,6^ In this study, we analyzed clinical and demographic factors associated with GT uptake in patients with cancer types for which there are guideline-based recommendations.

## Methods

We conducted a retrospective cohort study using electronic medical records (EMRs) from Columbia University Irving Medical Center, including patients with any-stage epithelial ovarian cancer or exocrine pancreatic cancer and metastatic prostate or breast cancer diagnosed between 2020 and 2023 (eFigure in Supplement 1). Informed consent was waived by the Columbia University Irving Medical Center institutional review board because the data were deidentified, in accordance with 45 CFR §46. All reported results adhere to STROBE guidelines.

The primary outcome, receipt of GT, was determined through review of the EMR. Baseline characteristics, including self-reported race and ethnicity, were analyzed using χ^2^ tests and *t* tests and/or analysis of variance. Univariable logistic regression was performed for each covariate to assess association with GT uptake. A multivariable logistic regression model was then constructed across all covariates regardless of univariant significance. Two-sided *P* < .05 was considered statistically significant for all analyses. Further methods can be reviewed in the eAppendix in Supplement 1.

## Results

Among 1670 evaluable patients (Table 1), the mean (SD) age was 67.9 (12.6) years. A total of 1096 patients (65.6%) underwent germline GT and 1033 (62.0%) received somatic testing. Only 335 patients (20.1%) were seen by a genetic counselor. Younger age, non-Hispanic White race and ethnicity, Jewish ancestry, tumor type of breast or ovarian cancer, family history of cancer, and receipt of somatic testing were all significantly associated with GT uptake (Table 2). Compared with patients with ovarian cancer, those with pancreatic cancer (odds ratio [OR], 0.36; 95% CI, 0.24-0.56) or prostate cancer (OR, 0.36; 95% CI, 0.21-0.61) were less likely to undergo germline GT. Compared with non-Hispanic White patients, individuals from other racial and ethnic groups were less likely to undergo testing (OR, 0.75; 95% CI, 0.58-0.97).

**Table 1.** Patients’ Demographic and Clinical Characteristics by Germline Testing Status

Characteristics	Patients, No. (%)			*P* value
	Total cohort (N = 1670)^a^	Germline genetic testing (n = 1096 [65.6%])^b^^,^^c^	No germline genetic testing (n = 574 [34.4%])^b^	
Age at diagnosis, mean (SD), y	67.9 (12.6)	66.2 (12.4)	71.0 (12.4)	<.001
Age group at diagnosis, y				
<40	78 (4.7)	65 (83.3)	13 (16.7)	<.001
40-49	261 (15.6)	197 (75.5)	64 (24.5)	
50-59	479 (28.7)	336 (70.1)	143 (29.9)	
60-69	530 (31.7)	319 (60.2)	211 (39.8)	
70-79	263 (15.8)	135 (51.3)	128 (48.7)	
≥80	59 (3.5)	44 (74.6)	15 (25.4)	
Sex				
Female	875 (52.4)	656 (75.0)	219 (25.0)	<.001
Male	795 (47.6)	440 (55.3)	355 (44.7)	
Race and ethnicity				
Asian or any other race, not otherwise specified	161 (9.6)	53 (32.9)	108 (67.1)	<.001
Hispanic	311 (18.6)	194 (62.4)	117 (37.6)	
Non-Hispanic Black	230 (13.8)	137 (59.6)	93 (40.4)	
Non-Hispanic White	791 (47.4)	560 (70.8)	231 (29.2)	
Unknown	177 (10.6)	97 (54.8)	80 (45.2)	
Jewish ancestry	205 (12.3)	158 (77.1)	47 (22.9)	<.001
Marital status				
Married	923 (55.3)	623 (67.5)	300 (32.5)	.13
Unmarried	678 (40.6)	433 (63.9)	245 (36.1)	
Other or unknown	69 (4.1)	40 (58.0)	29 (42.0)	
Tumor site				
Ovary	240 (14.4)	195 (81.3)	45 (18.8)	<.001
Breast	282 (16.9)	217 (77.0)	65 (23.0)	
Pancreas	694 (41.6)	481 (69.3)	213 (30.7)	
Prostate	454 (27.2)	203 (44.7)	251 (55.3)	
Year of diagnosis				
2020	457 (27.4)	279 (61.1)	178 (38.9)	.06
2021	459 (27.5)	304 (66.2)	155 (33.8)	
2022	437 (26.2)	304 (69.6)	133 (30.4)	
2023	317 (19.0)	209 (65.9)	108 (34.1)	
Family history of cancer				
Ovarian cancer	82 (4.9)	69 (84.1)	13 (15.9)	<.001
Breast cancer	420 (25.2)	331 (78.8)	89 (21.2)	<.001
Pancreatic cancer	142 (8.5)	113 (79.6)	29 (20.4)	<.001
Prostate cancer	218 (13.1)	157 (72.0)	61 (28.0)	.03
Somatic testing	1033 (61.9)	796 (77.1)	237 (22.9)	<.001
Seen by genetic counselor	335 (20.1)	316 (94.3)	19 (5.7)	<.001
Poly(adenosine diphosphate–ribose) polymerase inhibitor use	142 (8.5)	132 (93.0)	10 (7.0)	<.001

^a^ Percentages are shown by column.

^b^ Percentages are shown by row.

^c^ Results of germline genetic testing are as follows: pathogenic or likely pathogenic (202 patients [18.4%]), variant of uncertain significance (267 patients [24.4%]), negative (607 patients [55.4%]), and unknown (20 patients [1.8%]).

**Table 2.** Association Between Clinical and Demographic Characteristics and Germline Testing Status

Demographic or clinical factor	Univariable model, OR (95% CI)	*P* value	Multivariable model, OR (95% CI)	*P* value
Age at diagnosis (categories), y	0.97 (0.96-0.98)	<.001	0.98 (0.97-0.99)	<.001
Sex				
Female	1 [Reference]	NA	1 [Reference]	NA
Male	0.41 (0.34-0.51)	<.001	0.94 (0.67-1.33)	.74
Race and ethnicity (collapsed)				
Non-Hispanic White	1 [Reference]	NA	1 [Reference]	NA
All other racial and ethnic groups (Asian, Hispanic, Non-Hispanic Black, and any other)	0.69 (0.56-0.86)	.001	0.75 (0.58-0.97)	.03
Unknown	0.50 (0.36-0.70)	<.001	0.65 (0.44-0.96)	.03
Jewish ancestry	1.89 (1.34-2.66)	<.001	1.60 (1.08-2.36)	.02
Tumor site				
Ovary	1 [Reference]	NA	1 [Reference]	NA
Breast	0.77 (0.50-1.18)	.23	0.72 (0.46-1.13)	.15
Pancreas	0.52 (0.36-0.75)	<.001	0.36 (0.24-0.56)	<.001
Prostate	0.19 (0.13-0.27)	<.001	0.36 (0.21-0.61)	<.001
Marital status				
Unmarried	1 [Reference]	NA	1 [Reference]	NA
Married	1.18 (0.95-1.45)	.05	1.21 (0.94-1.54)	.14
Unknown	0.78 (0.47-1.29)	.19	0.86 (0.48-1.52)	.59
Year of diagnosis				
2020-2021	1 [Reference]	NA	1 [Reference]	NA
2022-2023	1.22 (0.99-1.49)	.06	1.19 (0.95-1.50)	.12
Family history of cancer				
Ovarian cancer	2.90 (1.59-5.29)	.001	1.99 (1.05-3.75)	.03
Breast cancer	2.36 (1.82-3.06)	<.001	1.83 (1.38-2.44)	<.001
Pancreatic cancer	2.16 (1.42-3.29)	<.001	1.83 (1.16-2.89)	.01
Prostate cancer	1.41 (1.03-1.93)	.03	1.72 (1.21-2.44)	.003
Somatic testing	3.77 (3.05-4.67)	<.001	3.32 (2.54-4.33)	<.001

Abbreviations: NA, not applicable; OR, odds ratio.

## Discussion

This study highlights several important trends in germline GT across 4 tumor types. Uptake of GT was similar or higher than prior reports, perhaps indicative of the adoption of National Comprehensive Cancer Network guidelines into clinical practice in recent years. When controlling for other variables, patients with pancreatic and metastatic prostate cancer had a 64% lower likelihood of germline GT compared with those with ovarian cancer.

Compared with non-Hispanic White patients, those from other racial and ethnic groups in our cohort had approximately 25% lower odds of GT uptake. These results are consistent with large-scale retrospective studies^5,6^ that reported significantly lower rates of GT in Black, Asian, and Hispanic patients with breast and ovarian cancer.

Despite the high uptake of GT, only 20.1% of patients in our cohort saw a genetic counselor, reflecting the ongoing transition toward oncologist-led testing. These findings may indicate increasing physician confidence with GT indications, panel selection, and result interpretation, including utilization of targeted therapies such as PARP inhibitors. We excluded patients with young-onset or operable high-risk ERBB2-negative breast cancer, for whom germline GT and adjuvant PARP inhibitors are indicated. Limitations include the single-center design at an urban academic medical center. Patients who may have received GT outside our institution may not have been captured in our data. Finally, the study period overlapped with the COVID-19 pandemic, during which the shift to telehealth may have exacerbated disparities in access to genetic testing and counseling. Additional interventions are necessary to address disparities in GT uptake, particularly among minoritized racial and ethnic groups, older patients, and those with prostate or pancreatic cancer.

## Notes

Supplement 1. **eAppendix.** Detailed Methods of This Study **eFigure.** Patient Flow Diagram

Supplement 2.
Data Sharing Statement

## References

[1] Daly MB, Pal T, Berry MP, . Genetic/familial high-risk assessment: breast, ovarian, and pancreatic, version 2.2021, NCCN clinical practice guidelines in oncology. J Natl Compr Canc Netw. 2021;19(1):77-102. doi:10.6004/jnccn.2021.0001 33406487

[2] Tung N, Ricker C, Messersmith H, . Selection of germline genetic testing panels in patients with cancer: ASCO guideline. J Clin Oncol. 2024;42(21):2599-2615. doi:10.1200/JCO.24.00662 38759122

[3] McCuaig JM, Thain E, Malcolmson J, Keshavarzi S, Armel SR, Kim RH. A comparison of patient-reported outcomes following consent for genetic testing using an oncologist- or genetic counselor-mediated model of care. Curr Oncol. 2021;28(2):1459-1471. doi:10.3390/curroncol28020138 33917963 PMC8167594

[4] Kurian AW, Li Y, Hamilton AS, . Gaps in incorporating germline genetic testing into treatment decision-making for early-stage breast cancer. J Clin Oncol. 2017;35(20):2232-2239. doi:10.1200/JCO.2016.71.6480 28402748 PMC5501363

[5] Kurian AW, Abrahamse P, Furgal A, . Germline genetic testing after cancer diagnosis. JAMA. 2023;330(1):43-51. doi:10.1001/jama.2023.9526 37276540 PMC10242510

[6] Lau-Min KS, McCarthy AM, Nathanson KL, Domchek SM. Nationwide trends and determinants of germline *BRCA1/2* testing in patients with breast and ovarian cancer. J Natl Compr Canc Netw. 2023;21(4):351-358.e4. doi:10.6004/jnccn.2022.7257 37015340 PMC10256435

