# Supplementary material for: Germline Genetic Testing in Patients With Solid Malignant Tumors
Source: JAMA Netw Open. 2025 Jul 23;8(7):e2522754. doi: 10.1001/jamanetworkopen.2025.22754 (PMC12287830; doi:10.1001/jamanetworkopen.2025.22754)
Supplement: Supplement 1. — eAppendix. Detailed Methods of This Study eFigure. Patient Flow Diagram [file jamanetwopen-e2522754-s001.pdf]

Supplemental Online Content

Fine ME, Estrin A, Michel A, et al. Germline genetic testing in patients with solid malignant tumors. *JAMA Netw Open.* 2025;8(7):e2522754.
doi:10.1001/jamanetworkopen.2025.22754

eAppendix. Detailed Methods of This Study

eFigure. CONSORT Diagram

This supplemental material has been provided by the authors to give readers additional information about their work.

© 2025 Fine ME et al. *JAMA Network Open*.

eAppendix. Detailed Methods of This Study

Study Design, Setting, and Participants

We conducted a retrospective cohort study using data curated from electronic medical records (EMR) at CUIMC in New York, NY among patients diagnosed with any stage epithelial ovarian cancer, any stage exocrine pancreatic cancer, metastatic prostate cancer, or metastatic breast cancer between 2020 and 2023. Any patient with at least one of these diagnoses was included for the initial chart review. Each record was reviewed to confirm diagnosis, staging at diagnosis, receipt of germline GT and results, somatic tumor genomic testing history, and family history of breast, ovarian, pancreatic, or prostate cancer. Patients were required to have documented visits with an oncologist in the CUIMC EMR to be included in our analysis. This study was approved by the institutional review board (IRB) at CUIMC and granted a waiver of informed consent.

The primary outcome of interest was receipt of germline GT (yes/no), based upon documentation in oncologist notes and scanned results. Among those who underwent germline GT, we also evaluated the result of genetic test including: benign/likely benign (B/LB), variant of uncertain significance (VUS), or pathogenic/likely pathogenic (P/LP) variant in any cancer predisposition gene, classified by the highest pathogenicity of the variants (*e.g.*, P/LP > VUS > B/LB). If there was information regarding genetic testing or genetic test results in the structured or unstructured data of the EMR, patients were classified as "tested." Patients with missing information on germline GT or a specific indication of "not tested" were classified as "not tested."

The main covariates of interest were age at cancer diagnosis, sex, self-reported race/ethnicity, marital status, primary tumor site, family history of breast, ovarian, pancreatic or prostate cancer, Jewish ancestry, year of diagnosis, and receipt of somatic testing. Targeted treatment with PARP-inhibitors and receipt of formal genetic counseling were also recorded, though not included as covariates with the

© 2025 Fine ME et al. *JAMA Network Open*.

primary outcome. The variables were extracted directly from the EMR or the New York-Presbyterian (NYP) Tumor Registry. Age at diagnosis was reported both categorically (<40, 40-49, 50-59, 60-69, 70-79, and 80+ years) and as a continuous variable. Sex was reported as male or female. Race was self-reported by patients and originally recorded in the EMR as American Indian or Alaska Native, Asian, Black or African American, Middle Eastern or North African, Multi-Racial, Native Hawaiian or Pacific Islander, Other Combinations Not Described, White, or Declined; ethnicity was recorded as Hispanic or Latino or Spanish Origin, Not Hispanic or Latino or Spanish Origin, or Declined. We combined race/ethnicity into Hispanic, non-Hispanic White, non-Hispanic Black, and Asian. The "Other" category included the remaining racial groups and was combined with Asian due to small numbers. Jewish ancestry, somatic testing on tumor tissue, targeted therapy with a PARP inhibitor, clinical encounter with a certified genetic counselor, and family history variables were reported as binary (yes/no). Marital status was reported categorically as married (including long-term domestic partners), unmarried (including divorced/widowed), and other/unknown. Primary tumor site was reported as breast, ovarian, pancreatic, or prostate. Year of diagnosis was reported as yearly categories for descriptive analyses but was collapsed into two-year categories for regression analyses (2020-2021, 2022-2023).

Statistical Analysis

Descriptive statistics were used to analyze baseline demographic and clinical characteristics for the overall population and stratified by germline GT status (yes/no). Chi-squared tests were used to compare dichotomous/categorical variables between those who were tested and those who were not; two-sample t-tests (or analyses of variance [ANOVA] for comparisons of more than two groups) were used to compare continuous variables. Fisher's exact tests were not required as all cells had at least five patients. All analyses were two-tailed and performed with a significance level of 0.05. Univariable

© 2025 Fine ME et al. *JAMA Network Open*.

models were run for each demographic/clinical characteristic to examine the association between that characteristic and undergoing germline GT. A multivariable model was also run including all variables, regardless of statistical significance in univariable analysis. SAS Studio was used to conduct all analyses.

© 2025 Fine ME et al. *JAMA Network Open*.

eFigure. CONSORT Diagram

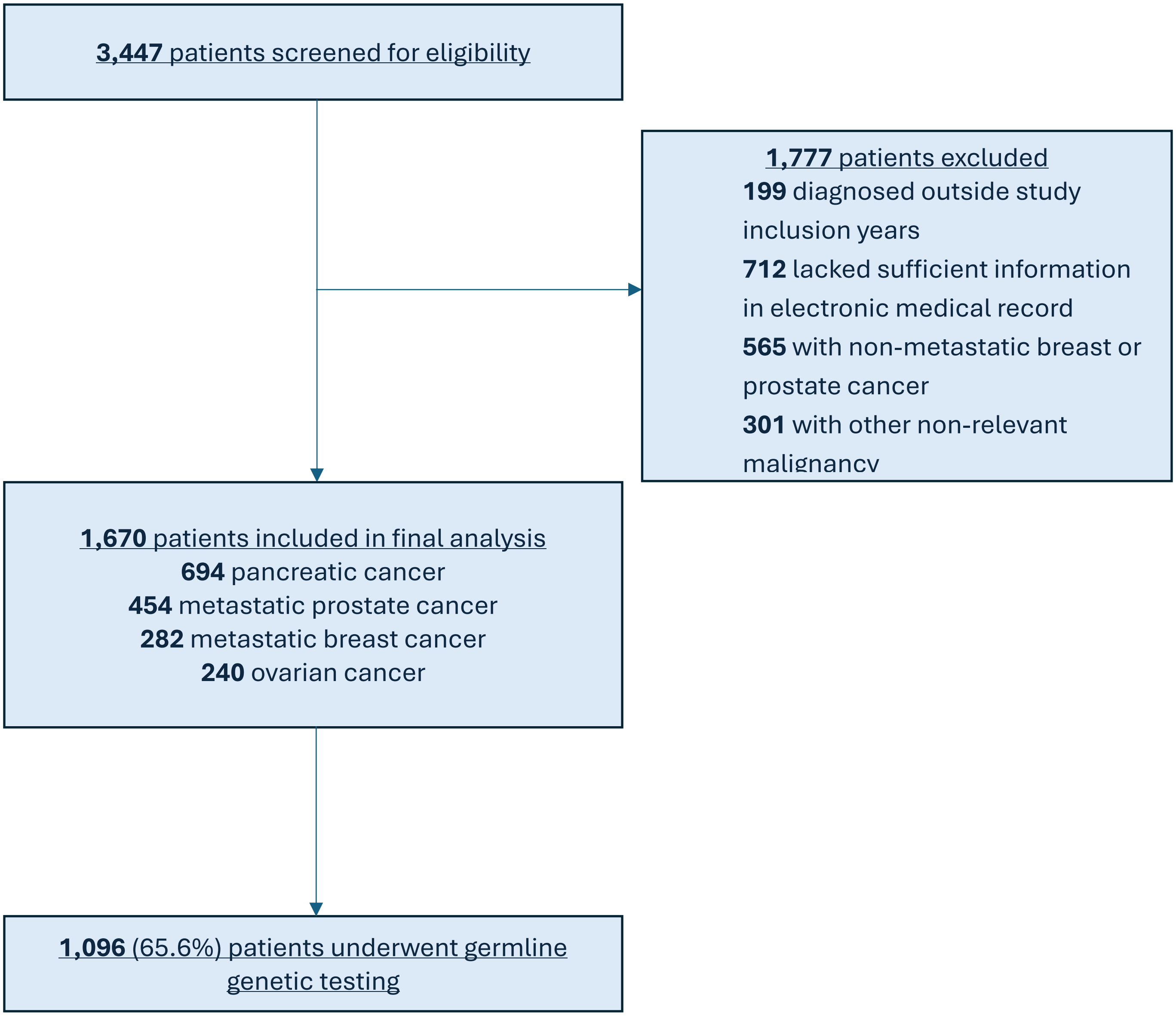

© 2025 Fine ME et al. *JAMA Network Open*.
