# Supplementary material for: Germline Genetic Testing in Patients With Solid Malignant Tumors
Source: JAMA Netw Open. 2025 Jul 23;8(7):e2522754. doi: 10.1001/jamanetworkopen.2025.22754 (PMC12287830; doi:10.1001/jamanetworkopen.2025.22754)
Supplement: Supplement 2. — Data Sharing Statement [file jamanetwopen-e2522754-s002.pdf]

Data Sharing Statement

Fine. Germline Genetic Testing in Patients With Solid Malignant Tumors. *JAMA Netw Open*. Published July 23, 2025. doi:10.1001/jamanetworkopen.2025.22754

Data
Data available: No

Additional Information
Explanation for why data not available: The data underlying this article will be shared on reasonable request to the corresponding author.
